# The ruminal microbiome associated with methane emissions from ruminant livestock

**DOI:** 10.1186/s40104-017-0141-0

**Published:** 2017-01-19

**Authors:** Ilma Tapio, Timothy J. Snelling, Francesco Strozzi, R. John Wallace

**Affiliations:** 1 Green Technology, Natural Resources Institute Finland, Jokioinen, Finland; 2 Rowett Institute of Nutrition and Health, University of Aberdeen, Foresterhill, Aberdeen, AB16 5BD UK; 3 PTP, Via Einstein - Loc. Cascina Codazza, 26900 Lodi, Italy

**Keywords:** Archaea, Methane, Microbiome, Rumen

## Abstract

Methane emissions from ruminant livestock contribute significantly to the large environmental footprint of agriculture. The rumen is the principal source of methane, and certain features of the microbiome are associated with low/high methane phenotypes. Despite their primary role in methanogenesis, the abundance of archaea has only a weak correlation with methane emissions from individual animals. The composition of the archaeal community appears to have a stronger effect, with animals harbouring the *Methanobrevibacter gottschalkii* clade tending to be associated with greater methane emissions. Ciliate protozoa produce abundant H_2_, the main substrate for methanogenesis in the rumen, and their removal (defaunation) results in an average 11% lower methane emissions in vivo, but the results are not consistent*.* Different protozoal genera seem to result in greater methane emissions, though community types (A, AB, B and O) did not differ. Within the bacteria, three different ‘ruminotypes’ have been identified, two of which predispose animals to have lower methane emissions. The two low-methane ruminotypes are generally characterized by less abundant H_2_-producing bacteria. A lower abundance of Proteobacteria and differences in certain Bacteroidetes and anaerobic fungi seem to be associated with high methane emissions. Rumen anaerobic fungi produce abundant H_2_ and formate, and their abundance generally corresponds to the level of methane emissions. Thus, microbiome analysis is consistent with known pathways for H_2_ production and methanogenesis, but not yet in a predictive manner. The production and utilisation of formate by the ruminal microbiota is poorly understood and may be a source of variability between animals.

## Background

Methane is a greenhouse gas (GHG) with a global warming potential 28-fold that of carbon dioxide [1]. Agriculture makes a significant contribution to total GHG production, with estimates varying according to country and calculation method [2]. Nonetheless, a global contribution of between 7 and 18% of total anthropogenic GHG emissions is generally accepted [2]. Ruminant production accounts for about 81% of GHG from the livestock sector (calculated from Hristov et al. [2]), 90% of which results from rumen microbial methanogenesis [3]. Ruminal CH_4_ production also represents a loss of energy (from 2 to 12% of gross energy intake [4]), which could in principle otherwise be available for animal growth or milk production. Lowering CH_4_ emissions therefore would benefit the environment and possibly the efficiency of livestock production. More than 87% of the CH_4_ produced by sheep has been estimated to be derived from the rumen [5], where a population of methanogenic archaea converts the H_2_ and CO_2_ produced by a complex community of ciliate protozoa, bacteria and anaerobic fungi to CH_4_ [6, 7]. A massive worldwide research effort has investigated various mitigation strategies. Changes in management practices can be simple and very effective [2], while feed additives that might inhibit H_2_ production, provide an alternative metabolic H sink or inhibit the archaea themselves offer opportunities beyond those straightforward management changes [6–11]. Other opportunities include chemogenomics and immunization [12–14]. One strategy that is foremost in several investigations is genetic selection of the livestock. If we can demonstrate that persistently different CH_4_ emissions in different animals [14–16] can be explained by their individual ruminal microbiomes, and that the characteristic is heritable, it should be possible to select future generations of ruminants that have intrinsically lower CH_4_ emissions. All the strategies potentially involve changing the ruminal microbiome. The aim of this short review is to assess our current understanding of the role of different members of the microbiome in determining the extent of methanogenesis in the rumen.

### The rumen microbial community

The rumen is home to a vast array of ciliate protozoa, anaerobic fungi, anaerobic bacteria and archaea. The protozoa can comprise up to half the rumen microbial biomass [17, 18], the fungi were originally estimated to be about 8% of the biomass [19] but may reach 20% in sheep [20], the archaea comprise 0.3–4% [21] and the bacteria form the remainder, typically the largest component of the microbial biomass. Our present understanding of ruminal microbiology was built initially upon a few epoch-changing advances made many years ago: Gruby & Delafond’s [22] microscopic observations of protozoa; Hungate’s [23] appreciation of the anaerobic nature of the rumen that led to new, truly anaerobic culture techniques for the bacteria; Orpin’s [24] realization that some flagellate protozoa were in fact zoospores of anaerobic fungi, until then a contradiction in terms. The isolation and study of pure cultures was and remains invaluable in understanding the likely role of different species of bacteria, protozoa or fungi in the overall fermentation. Drawbacks of cultivation techniques are that only a very small number of samples can be tested, and that they suffer from bias, whereby the composition of the growth medium, generally too rich, determines which species can grow [25]. Development of molecular techniques, based mainly on ssu rRNA gene and intergenic spacer sequence (for the fungi) analyses, opened new opportunities in rumen research. Cloning and sequencing provided community analyses that were not prone to the biases imposed by cultivation techniques, although different bias was introduced by other factors, like storage conditions [26], the differential efficiency of DNA extraction from different species and amplification bias [27–29]. Related techniques for microbiome analysis quickly followed (DGGE, TGGE, T-RFLP, ARISA). Quantitative PCR and FISH enabled microbial groups or species to be quantified [30]. Now, metagenomic sequencing enables rapid community analysis to be carried out, without the cultivation bias or variation associated with primer selection or PCR amplification irregularities [25, 31]. The problem of DNA extraction remains, however, and databases are relatively weak where ruminal organisms are concerned [32]. Nevertheless, if we can use this approach to determine how the functional activity of the rumen microbial community influences methane emissions, the knowledge should enable strategies to decrease the environmental impact of livestock agriculture. Furthermore, it might be expected to improve animal production efficiency.

### Ruminal community analysis relating to methane emissions

#### Archaea

There are two main routes for methanogenesis in the rumen, both carried out by archaea. The hydrogenotrophic pathway converts H_2_ and CO_2_ produced by the protozoa, bacteria and fungi to CH_4_ [3, 6]. It is usually assumed that formate, which can be used by all the most abundant ruminal archaea, is equivalent to H_2_ + CO_2_, so formate is included in the hydrogenotrophic category [21, 33]. A second category of substrate for methanogenesis is methyl groups, such as those present in methylamines and methanol [34, 35]. Methylamines are derived from glycine betaine (from beet) and choline (from plant membranes), while methanol is derived from the hydrolysis of methanolic side-groups in plant polysaccharides. The most common hydrogenotrophic archaea are from the genus *Methanobrevibacter*, which has been divided into two subgroups, one known as the SGMT clade (*Mbb. smithii*, *Mbb. gottschalkii*, *Mbb. millerae* and *Mbb. thaueri*), the other (RO) clade comprising principally *Mbb. ruminantium* and *Mbb. olleyae* [21, 36]. Other significant hydrogenotrophic genera include *Methanosphaera, Methanimicrococcus* and *Methanobacterium.* The less abundant methylotrophs (Methanosarcinales, *Methanosphaera*, Methanomassiliicoccaceae) can use methylamines and methanol, and there are archaea (Methanosarcinales) that produce methane via the aceticlastic pathway (reviewed in Morgavi et al. [7]). Rumen methanogenic archaeal diversity is restricted to four orders [21] and is highly conserved across 32 ruminant species collected worldwide [32].

**Fig. 1:**
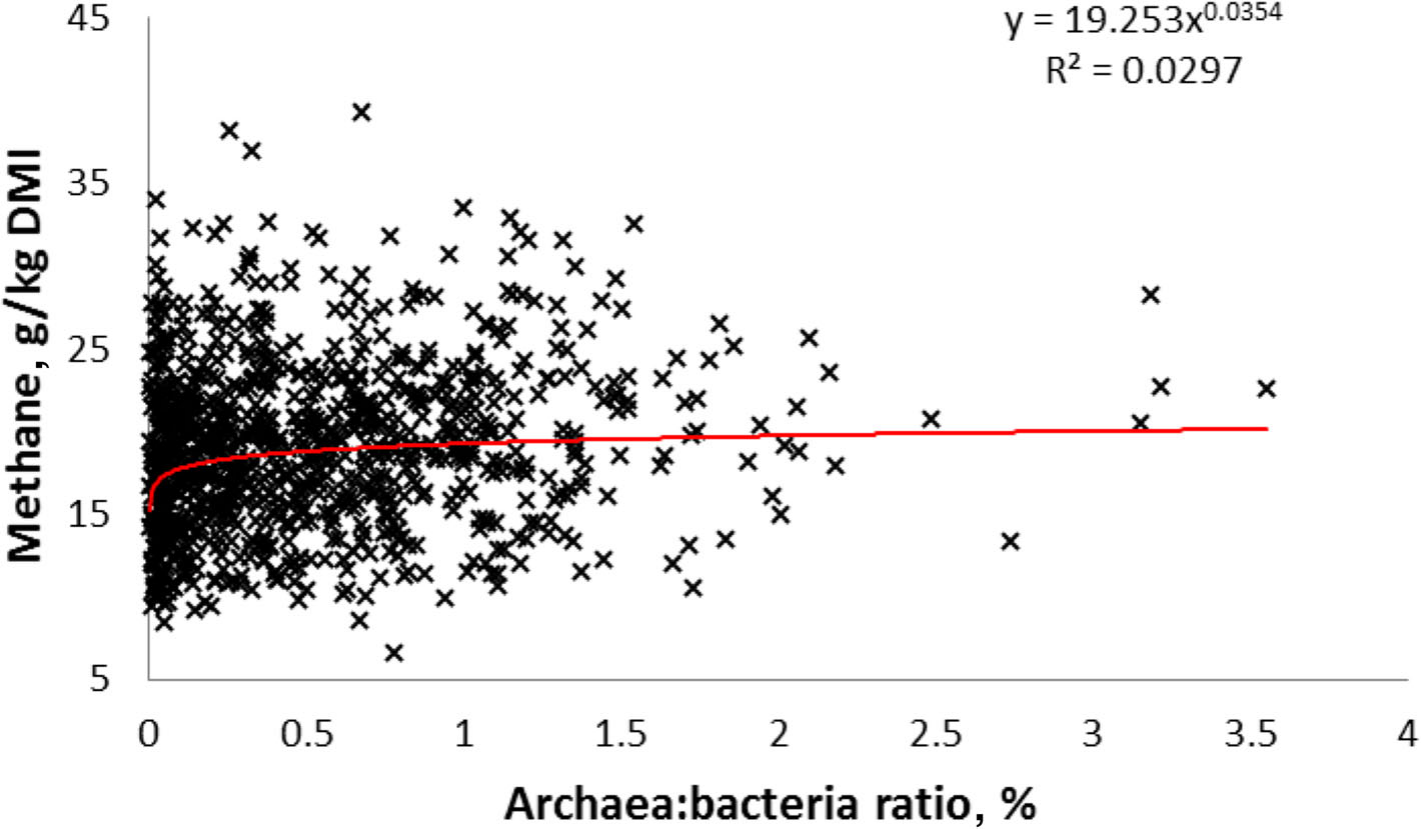
Archaea:bacteria relative abundance in relation to methane emissions, preliminary data from the 1000-cow RuminOmics project. Dairy cows on different farms throughout Europe received grass or maize silage:concentrate diets of similar nutrient composition. Feed intake was measured either directly or calculated from faecal long-chain hydrocarbons. Samples of rumen contents were removed by stomach tube and DNA was extracted by the Yu & Morrison method [110]. Abundances were calculated from qPCR of 16S rRNA genes using universal primers for archaea and bacteria

Intuitively, archaea should be the microbial group most closely correlated with methane emissions. However, some studies have shown no such correlation with their overall abundance while in others the correlation has been weak. Morgavi et al. [37], Zhou et al. [38], Danielsson et al. [39] and Danielsson [40] found no correspondence between the numbers of methanogens and methane emissions from dairy cows when measured using metagenomics and qPCR techniques. Kittelmann et al. [41] and Shi et al. [42] formed a similar conclusion in sheep. A weak correlation between archaeal abundance relative to bacteria was found in beef steers [43] but none was found with dairy cows in the RuminOmics project [http://www.ruminomics.eu/] when expressed as the archaea:bacteria ratio (Fig. 1). Shi et al. [42] also observed that archaeal gene expression rather than gene abundance was correlated to methane emissions from individual sheep. It is easy to see why gene expression might be a useful proxy for methanogenesis in a static system like soil [44], but less so in a flowing system like the rumen, where for physiological reasons biomass must be directly correlated to gene abundance unless other processes, such as uncoupled CH_4_ production occur [45].

Given the high variability of the relationship with overall archaeal abundance, it may be that the composition of the archaeal community rather than just its size may have greater significance with regard to methane emissions. Zhou et al. [38], Danielsson et al. [39], Shi et al. [42] and Danielsson [40] all found a positive correlation between the relative abundance of *Methanobrevibacter* SGMT clade and methane emissions. Danielsson [40] interpreted this correlation in terms of different affinities for H_2_ in the two groups, with the SGMT clade possessing methyl coenzyme M reductase isozymes McrI and McrII [12], which enables the archaea to utilise H_2_ at higher concentrations, against the RO clade that possess only McrI [3, 12]. The dynamics of the of the archaeal community composition and thus the efficiency of H_2_ utilization would in turn would be a consequence of differing H_2_ production by different bacteria [33, 41] and presumably also protozoal and fungal communities. Furthermore, the proportion of *Methanosphaera* spp. in total archaea was negatively associated with methane production in sheep [41], although not in beef cattle [46]. Thus, differing methane emissions are at least partly due to varying relative abundances within the community of methanogenic archaea.

Other observations regarding the archaeal community, sometimes called the archaeome, include those of Pitta et al. [47], who found that archaeal abundance increased in steers suffering frothy bloat, and Pei et al. [48], who discovered archaea associated with the rumen epithelium. In the former case, the CH_4_ content of the gas was not measured, so it is unclear the impact the bloat would have on methanogenesis. In the latter, the finding was surprising because the rumen wall is considered to be an aerobic/anaerobic interface, and the relative abundance of O_2_ might be considered to suppress the growth of the extremely O_2_-sensitive methanogens. In fact, one might have possibly expected CH_4_ oxidisers to be present, in spite of their absence from the deep ruminal digesta [49].

#### Ciliate protozoa

**Fig. 2:**
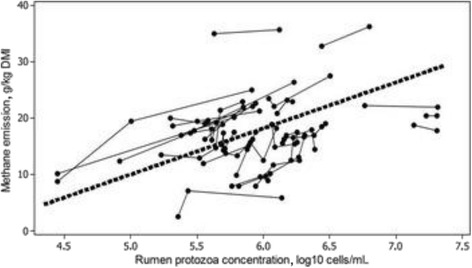
Relationship between methane emission and rumen protozoa concentration in a meta-analysis of 28 different experiments. The *black dashed* line represents the average within-experiment relationship. Reproduced from [56] with permission

Ruminal ciliates are intimately involved in methanogenesis, partly via their abundant H_2_ production [50] and, taking advantage of this, their associated methanogens, which are found both as intracytoplasmic commensals and on the exterior surface of the protozoa [3, 18, 51–53]. Several studies suggested a correlation between the abundance of protozoa and methane emissions (collated in [18, 54, 55]), while others do not [37, 43]. Guyader et al. [56] conducted a meta-analysis containing 28 experiments and 91 treatments. This meta-analysis showed a linear positive relationship between log_10_ protozoal numbers and methane emissions expressed per unit DMI. An *r* = 0.96 showed that there is indeed a reasonably strong relationship (Fig. 2).

Defaunation (the removal of the ciliates from the rumen) has therefore been investigated in relation to methane production. Although in some cases the results of defaunation on CH_4_ emissions have not been encouraging [57–60], Newbold et al. [18] carried out a meta-analysis of defaunation studies and concluded that CH_4_ was decreased on average by 11%. Despite the lower CH_4_ production, the total archaeal abundance was not significantly decreased in the Newbold et al. meta-analysis, suggesting that the archaeal community in defaunated animals may have a lower CH_4_-emitting specific activity than that of the protozoa-associated community.

As with the archaea, the questions then revert to whether some individual protozoal genera or species, and their associated archaea, are more linked with methanogenesis than others. In general, the protozoa harbour an archaeal population that, like the general archaeal community, is dominated by *Methanobrevibacter* spp. [61–64], although differences were observed in the abundance of different archaea found in the protozoa and in the non-associated archaea [18, 61, 65] that might lead to different methanogenic specific activities in the two populations. Furthermore, archaeal colonisation abundance may differ between different protozoal species [51] and each may be associated with different predominant archaeal genera/species. Holotrichs in particular had an archaeal community that differed from entodiniomorphid protozoa [53]. Larger ciliates appear to be more heavily colonized by methanogens than smaller ciliates [53, 66], and also by bacteria, suggesting that there is not a selective colonisation by archaea [53]. The lower metabolic activity in terms of H_2_ production of the larger protozoal species per unit biomass [50, 54, 58] presumably explains that smaller protozoa, and their associated archaea, will be relatively more active in methanogenesis than larger species. Indeed, in vitro studies indicated that the smaller *Entodinium* spp. were more associated with methane production than larger species like *Polyplastron multivesiculatum* [50, 58]. In vivo studies are inconsistent, however. Refaunation experiments indicated that the abundance of *Entodinium* spp. [67, 68] or holotrichs [68] correlated with higher methane emissions. A large amplicon sequencing study in sheep nevertheless found no relationship between the relative abundance of different ciliates and methane emissions [41]. Furthermore, ciliate communities fall into a small number of types (A, AB, B and O [69]) depending on interactions, principally inter-species predation. Despite the large differences in relative abundance of different protozoa types in the different community types, methane emissions could not be correlated with protozoal community structure [70]. The varying colonisation by archaea depending on the time after feeding [71] is another confounding factor in trying to evaluate the role of protozoa in methanogenesis.

#### Bacteria

**Fig. 3:**
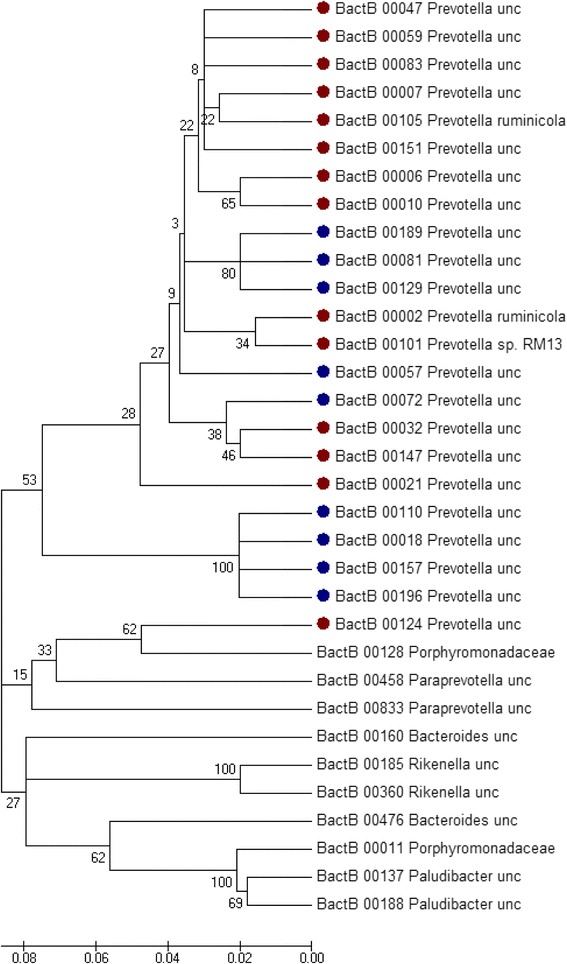
Neighbor Joining tree of *Prevotella*-like OTUs that had a negative (blue dots) or positive (*red dots*) relation to methane (expressed in terms of g methane/kg DMI) in the 1,000-cow RuminOmics project. Multiple alignment was done using MUSCLE [111]. The Neighbor Joining tree was constructed using p-distance and pairwise-deletion parameters. The tree was resampled 1,000 times and bootstrap values are indicated. The linearized tree was computed using MEGA v5.1 [112] by using most abundant Bacteroidales OTUs to create an “outgroup”

Ruminal bacteria form the most diverse group within the rumen, capable of utilizing fibre, starch, protein and sugars [72]. Among numerous bacterial phyla found in different studies, Firmicutes, Bacteroidetes and Proteobacteria are the most abundant [32]. Fibrolytic bacteria, especially cellulolytic *Ruminococcus* and several *Eubacterium* spp (Firmicutes), are well studied H_2_ producers. On the other hand, the prominent cellulolytic genus, *Fibrobacter*, does not produce H_2_, while Bacteroidetes are net H_2_ utilizers [72]. Microbiome analysis has identified three different ‘ruminotypes’ that seemed to be associated with variations in methane production by sheep [41]. The low-CH_4_ production ruminotype Q was characterised by high relative abundances of the propionate-producing *Quinella ovalis*. Low-CH_4_ ruminotype S had higher abundances of lactate- and succinate-producing *Fibrobacter* spp., *Kandleria vitulina*, *Olsenella* spp., *Prevotella bryantii*, and *Sharpea azabuensis*. The high-CH_4_ production ruminotype H had higher relative abundances of species belonging to *Ruminococcus*, other Ruminococcaceae, Lachnospiraceae, Catabacteriaceae, *Coprococcus*, other Clostridiales, *Prevotella*, other Bacteroidales, and Alphaproteobacteria. The overall interpretation would be that methane emissions depend on the abundance of the H_2_-producing bacteria present; a corollary to this is the observation that chemical inhibition of methanogenesis in goats led to increases in the abundance of H_2_-consuming *Prevotella* and *Selenomonas* spp*.* [73]. Proteobacteria were 4-fold less abundant (2.7 vs. 11.2% of bacteria) in high emitting beef cattle [46] and a similar finding was made in dairy cows [40]. The dominant family among Proteobacteria was Succinivibrionaceae. This finding seems to parallel the high numbers of Succinivibrionaceae in the Tammar wallaby [74], which, like the ruminant, is a herbivorous foregut fermenter. It produces only about one-fifth of the methane per unit of feed intake of ruminants, which is attributed to the large community of Succinovibrionaceae. An intriguing additional observation common to these studies [40, 41] was that within different *Prevotella* OTUs, some were correlated with a high CH_4_ phenotype, while others were associated with low emissions. The different OTUs seem to cluster together (Fig. 3), suggesting functional versatility within the *Prevotella* genus. Further investigation of the phenotypes of these dominant ruminal bacteria is needed, which may well provide clues for future exploitation, particularly as some *Prevotella* are reported to produce formate [72].

In a dairy cattle study [75] with two CH_4_-mitigating feed additives, grapemarc and a combination of lipids and tannins, it was found that the microbiome differed from the control diet in a similar way. *Faecalibacterium prausnitzii* was over-represented in the low- CH_4_ diets, and other microbiome markers that could be predictive of low-CH_4_ phenotypes were identified. *F. prausnitzii* is a bacterial species that is abundant in the human colon [76] but is seldom mentioned in the context of the rumen. It may prove a useful marker, but it is not obvious how its properties could be mechanistically connected to the low-CH_4_ phenotype.

#### Anaerobic fungi

The anaerobic fungi, like the protozoa, produce abundant amounts of H_2_, along with CO_2_, formate and acetate as metabolic end products [77]. Six fungal genera have been detected in the rumen but recent molecular research suggests existence of several new taxa [78], with functions still to be understood. Methanogens are found in close association with fungal hyphae [79]. Although there is reason to suppose that fungal abundance might be related to methane emissions, reports are few. Kittelmann et al. [41] noted no difference in fungal community structure in relation to methane emissions from sheep. In the RuminOmics project, however, preliminary results suggest that two fungal species, *Caecomyces communis* and *Neocallimastix frontalis*, are negatively related to methanogenesis (*r* = -0.50 and -0.45, *P* < 0.001; R.J. Wallace et al., unpublished]. The meta-analysis of Newbold et al. [18] noted that one of largest effects of defaunation, which leads to lower CH_4_ production, was a decrease in fungal abundance. Whether this decrease is a major or direct cause of lower CH_4_ production in defaunated animals is unclear.

### General considerations on variations in methanogenesis and the microbiome

#### Contribution of non-hydrogenotrophic methanogenesis

The main substrates for methanogenesis in the rumen are known to be H_2_ + CO_2_, formate and compounds containing methyl groups like the methylamines and methanol [21]. In the reviews already mentioned here, formate and H_2_ + CO_2_ are usually considered to be equivalent as substrates for methanogenesis and formate is not treated separately. Formate feeds directly into the methanogenesis pathway at the very beginning via formate dehydrogenase [80]. Hungate et al. [81] estimated that 18% of methane was formed via formate rather than H_2_ + CO_2_. Yet there are some important aspects of formate metabolism about which our understanding is incomplete. The relationship between bacterial abundances from microbiome estimates, above, was discussed in relation to whether bacteria form H_2_, as in other analyses [33, 41, 43, 46], with little indication about formate producers. There is a large uncertainty about bacterial formate production, reflected in the summary tables of Stewart et al. [72]. Although many species produce some formate, precise amounts are not known and therefore the importance of this production is difficult to estimate. Perhaps the Hungate 1000 collection (www.rmgnetwork.org/hungate1000.html) could be used as a resource to make such measurements. At present, the Hungate 1000 project has its emphasis on strengthening genetic databases [3], but much phenotypic information is being collected alongside the main thrust of the project. Assessing bacterial formate production is further complicated by the knowledge that co-culture experiments demonstrate that the metabolism of some bacteria and fungi grown in the presence of methanogens can be pulled in the direction of H_2_ or formate production [82–85], so it is very difficult to be sure what the role of different species might be in the mixed rumen community. And perhaps most crucially, methanogenesis is not the sole fate of formate in the rumen. Hungate et al. [81] noted formate utilisation in the absence of methanogenesis, presumably by bacteria. Species like *Wolinella succinogenes* use formate as an energy source [72]. So, although it is usually stated that ruminal archaea utilise either H_2_ + CO_2_ or formate [3], it is unclear whether they are indeed equivalent for different archaea. For example, in co-cultures between rumen anaerobic fungi and three methanogens, all the methanogens used H_2_ but formate was only utilised simultaneously by *M. smithii* [86]. The differential expression of formate dehydrogenase was one of the largest differences between high- and low-emitting sheep [42]. The formate dehydrogenase of *M. ruminantium* M1 was induced by co-culture with the formate-producing *Butyrivibrio proteoclasticus* [12]. Thus there are several reasons to conclude that thinking about formate as a substrate in the context of microbiomes differing in their methanogenic activity might prove fruitful. Furthermore, despite the emphasis on H_2_ produced by ciliate protozoa, the quantity of formate produced seems to be many times greater than H_2_ [65].

The methylamines and methanol are methyl donors for methanogenesis by methylotrophic archaea, as described above. Their contribution to methanogenesis will depend to some extent on the concentration of methylamines in the diet [34, 35]. But how efficient is the process? Are methylamines converted quantitatively to CH_4_, and are methylamine, dimethylamine and trimethylamine equivalent in that respect? It is possible that variation in CH_4_ emissions between individual animals on some diets may be due to different efficiencies whereby methylamines are released from feed materials and converted to CH_4_.

One of the more surprising findings in the *Mbb. ruminantium* M1 genome was the presence of three genes encoding alcohol dehydrogenase [12]. It has been demonstrated that ethanol can be used as a C source, but not as sole C source [3]. Thus, the availability of ethanol from bacterial fermentation may influence the dependence of archaea on methanogenesis for ATP production, and therefore affect the quantity of CH_4_ produced.

#### Influence of diet and mitigation measures

An important principle underlying this review is that some microbiomes lead to different CH_4_ emissions when other factors remain constant. Thus, key members of the microbiome leading to high or low emissions should be able to be identified. In the RuminOmics project, all dairy cows received diets that were as nutritionally similar as was possible given the different locations. Only by keeping as many other factors as possible unchanged will it be possible to dissect the role of different members of the microbial community in determining low- and high-emitting individuals. It should be noted here that we have chosen to express CH_4_ production in terms of DMI, for the simple reason that it makes it easier to identify a low-CH_4_ microbiome rather than a microbiome that forms less CH_4_ only because the host animal eats less.

The results of microbiome analysis so far were expected in some respects, in the sense that diets high in starch content are known to lead to lower methane emissions, because starch utilising bacteria tend to produce less H_2_ than others, for example [33, 72]. In a similar way, the changed fermentation stoichiometry linked with methane emissions is a very long established observation [87, 88]. New questions have been highlighted regarding different species associated with high and low CH_4_ emissions under similar conditions. Unexpected correlations have been found. But many questions remain. It is also worth noting that widely different taxa may have similar metabolic activities [89], so there are several different microbiota that could lead to similar metabolic properties.

Mitigation measures have been described comprehensively elsewhere [2, 3, 6–10]. Perhaps the most promising of these is 3-nitrooxypropanol, a molecule obtained rationally by its structural similarity to methyl-CoM [90–92]. As yet we do not know the full implications of 3-nitrooxypropanol, but encouragement can be obtained that the concern that H_2_ accumulation might inhibit overall fermentation does not seem to be such a problem as was suggested by some in vitro experiments [33, 93]. It is also worth noting that a 50% reduction in the growth rate of methanogens would be sufficient to cause their washout from the rumen [3, 33]. Complete inhibition of growth is therefore not necessary.

#### Methane and feed efficiency

CH_4_ production and feed efficiency are linked, in the sense that a low feed efficiency, expressed as residual feed intake (RFI), is accompanied by lower CH_4_ production [94–96]. The reverse does not apply, however, as has been found in dairy cows in the RuminOmics project. The findings that the abundance of certain *Prevotella* changes according to feed efficiency in beef cattle [97, 98] and many other taxa change in abundance [98] further emphasises our need to understand the role of *Prevotella* and its different biotypes on ruminal fermentation and methanogenesis. Shabat et al. [99] discovered that *Megasphaera elsdenii* was more abundant in low-efficiency cows, as were genes of the acrylate pathway, used by *M. elsdenii* in propionate formation. The explanation for lower efficiency was that *M. elsdenii* introduced a type of futile cycle in the production and subsequent utilisation of lactate, an energetically inefficient process.

#### The influence of the host animal

Many researchers believe, and some studies are beginning to show, that the host animal exerts a controlling effect on its own gut microbiota [100–102]. The mechanism could conceivably be at a molecular level, perhaps via complex interactions with receptors in the rumen wall [103, 104] or antibodies in saliva [3, 105, 106]. More likely, however, is that the physical structure and dynamics of gut digesta are different in different animals. Goopy et al [15] found that lower methanogenesis in sheep was heritable and accompanied by the animals’ having smaller rumen volumes and therefore altered fluxes of nutrients through the tract. This would have the effect that less feed would be fermented in the rumen, leading to lower methanogenesis. Variations in saliva production could lead to a similar result [107]. Both would likely influence the ruminal microbiome. Therefore, caution should be exercised in interpreting microbiome analyses – the changed microbiome may be associated with, but not cause, a decrease in methanogenesis.

Ross et al. [108] found good correlations between CH_4_ emissions and the broad characteristics of the microbiome. Now, metagenomics has shown that the abundance of certain groups of microbial genes can be highly predictive of CH_4_ emissions [46, 109] and feed efficiency [99]. For example, 20 microbial genes explained 81% of variation in CH_4_ emissions from beef cattle, while 49 genes explained 86% of variation in RFI [109]. Furthermore, the animal’s genetic background was a factor in determining these gene abundances [109]. This is the early phase of what is sure to be a fertile area in which animal-microbiome-emissions can be delineated by metagenomics profiling, and animal breeding based on these gene abundances may lead to animals with lower CH_4_ emissions.

## Conclusions

Recent large scale projects such as the Global Rumen Census, the Hungate 1000 and RuminOmics, from which some preliminary results are presented here, have provided new depth of insight into the composition and function of the rumen microbial community. By revealing the some of the relationships between the microbiome and the animal phenotype, they have shown how understanding the role of the rumen microbiota can help in the efforts to reduce the environmental impact of livestock agriculture, in particular with the amelioration of greenhouse gas emissions. The archaea have been the main target for research, being directly associated with methane production in the rumen. However, other major microbial groups such as the ciliate protozoa, the anaerobic fungi, Succinovibrionaceae and *Prevotella*, among others, have shown to be associated with both high and low methane production. The results illustrate that there are basic phenotypic characteristics, such as formate metabolism, that are insufficiently understood. When placed in the context of the many as yet uncultivated microbial species of the rumen, it becomes clear that the powerful tool of molecular analysis must be accompanied by cultural and metabolic/phenotypic analysis if we are to truly understand the relation between the ruminal microbiome and methanogenesis.

## Abbreviations

ARISA: Automated ribosomal intergenic spacer analysis
DGGE: Density gradient gel electrophoresis
FISH: Fluorescence in vitro hybridization
qPCR: Quantitative polymerase chain reaction
TGGE: Temperature gradient gel electrophoresis
T-RFLP: Restriction fragment length polymorphism
